# Weaning Induced Gut Dysfunction and Nutritional Interventions in Nursery Pigs: A Partial Review

**DOI:** 10.3390/ani11051279

**Published:** 2021-04-29

**Authors:** Xiaoyuan Wei, Tsungcheng Tsai, Samantha Howe, Jiangchao Zhao

**Affiliations:** Department of Animal Science, Division of Agriculture, University of Arkansas, Fayetteville, AR 72701, USA; xw010@uark.edu (X.W.); ttsai@uark.edu (T.T.); smhowe@uark.edu (S.H.)

**Keywords:** weaning pigs, gut microbiota, supplements, growth performance, intestinal balance

## Abstract

**Simple Summary:**

The beneficial and functional roles of the gut microbiota in maintaining host health are becoming clearer. In general, the swine intestinal bacterial population is predominated by two phyla, Firmicutes and Bacteroidetes, followed by Proteobacteria, Actinobacteria, and Spirochaetes, among others. This general profile is relatively stable, although variations exhibit below the phylum level when the gut microbiota experiences dysbiosis. At weaning, dietary and environmental changes often result in gut microbiota dysbiosis, which is frequently associated with post-weaning diarrhea and enteric infections. A healthy gut microbiota possesses specific capacities involving nutrient metabolism, immunomodulation, controlling inflammation, and maintaining intestinal barrier function and structure. Manipulating the gut microbiota through diet is a useful strategy to attenuate weaning stress and restore microbiota homeostasis. Understanding the interaction between feed additives and gut microbiota would facilitate the development of effective feeding strategies to improve the gut health, growth performance, and well-being of nursery pigs. This review summarizes certain feed additives, which are used to modulate gut microbiota with some success.

**Abstract:**

Weaning is one of the most stressful events in the life of a pig. Unsuccessful weaning often leads to intestinal and immune system dysfunctions, resulting in poor growth performance as well as increased morbidity and mortality. The gut microbiota community is a complex ecosystem and is considered an “organ,” producing various metabolites with many beneficial functions. In this review, we briefly introduce weaning-associated gut microbiota dysbiosis. Then, we explain the importance of maintaining a balanced gut microbiota. Finally, we discuss dietary supplements and their abilities to restore intestinal balance and improve the growth performance of weaning pigs.

## 1. Introduction

Weaning is a stressful period for pigs due to the challenge of adapting to solid feed, maternal separation, transportation, and establishing a social hierarchy [1]. Weaning stress could disrupt intestinal barrier integrity and disturb the ecological balance of the gut microbial community [2,3,4,5]. Disrupting the balance of the gastrointestinal (GI) tract microbiota, known as dysbiosis, favors pathogen colonization on epithelial cells, leading to local inflammation [6]. Moreover, the immature immune system of newly weaned pigs can exacerbate their inflammatory response and cause them to be more susceptible to cytokine storms and sepsis [7,8,9].

The gut microbial community is largely impacted by diet [10]. The digestive system of newly weaned pigs is inefficient at utilizing nutrients from plant-based feed ingredients [11,12]. At weaning, the diet changes from a highly digestible liquid milk to a solid, less digestible nursery diet, which facilitates structural remodeling of the GI tract morphology and microbiota [13]. During this transition period, feed efficiency and nutrient digestibility are typically low. Due to the ban on antibiotics as growth promoters in animal feed, nutritional strategies have been applied to minimize the adverse effects of weaning stress. Accumulating evidence suggests that the benefits of certain feed additives on animal growth performance are partially through gut microbiota modulation [14,15,16]. Nowadays, the advent of next-generation sequencing (NGS) technology provides insight into the diverse and complex gut microbiota, which remarkably expanded our knowledge regarding how the entire gut microbiota evolves under the influence of feed additives.

In this review, we introduce the relationship between gut microbiota dysbiosis and weaning. Then, we briefly discuss the importance of maintaining a healthy gut. Finally, we summarize available information on the efficacy of nutritional interventions such as zinc, peptides, organic acids, and probiotics on the gut ecosystem integrity of weaned pigs. This review can assist future researchers and industries in developing novel strategies to modulate the swine gut microbiota through diet with the ultimate goal of improving growth performance and gut health.

## 2. Weaning Stress Disturbs the Gut Microbiota and the Importance of a Healthy Gut

### 2.1. Intestinal Dysfunction Induced by Weaning Stress

**Figure 1:**
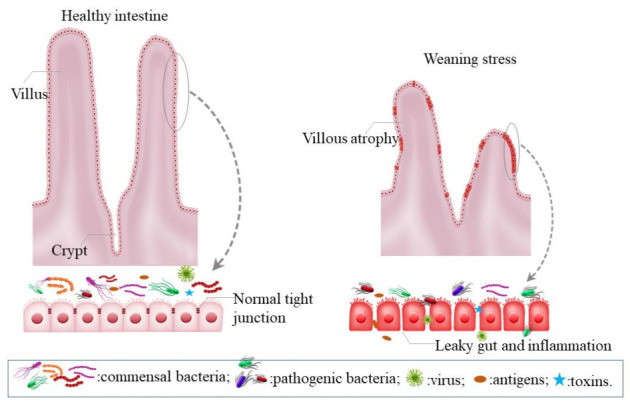
Negative impacts of weaning stress on swine gut health.

During the weaning process, adverse changes occur in the GI tract, such as villous atrophy, crypt hyperplasia, intestinal inflammation, and increased gut permeability caused by the disruption of tight junctions [17,18,19,20]. A healthy gut harbors a highly diverse community of commensal bacteria and possesses a barrier function that helps to prevent pathogenic microbes from entering the body. However, a decreased microbiota diversity and a leaky gut, caused by weaning stress, would allow pathogenic bacteria, toxins, and harmful antigens to enter the host and cause diseases (Figure 1). These main intestinal characteristics in weaned piglets have recently been reviewed [1,21,22,23] and will not be reiterated here.

Nowadays, it is widely recognized that dietary and environmental changes at weaning contribute to the modification of the gut microbiota, which may be associated with post-weaning diarrhea and enteric infections [13]. The gut microbiota community is a complex ecosystem and is considered an “organ”, producing various metabolites with many potential functions [23,24]. Microorganisms colonize the sterile gastrointestinal tract immediately after birth and take several weeks to fully develop [25,26]. Sow’s milk is the primary nutrient source for both newborn piglets and their microbiota. Thus, it imposes a selection on the gut microbial community, allowing some microbes to dominate the gut ecosystem [27,28,29,30]. The initial predominant colonizers, such as *Escherichia coli* (*E. coli*) and *Streptococcus* spp., could facilitate the colonization of later species [10,31]. For example, *E.coli* depletes oxygen in the gut, creating an anaerobic environment for late-arriving *Bacteroides* spp. [32]. Milk also provides antimicrobial factors, including secretory immunoglobulin A (IgA), lysozyme, and lactoferrin, which promote intestinal homeostasis [33,34].

Weaning stress, however, disrupts gut microbiota homeostasis, providing pathogenic bacteria an opportunity to multiply, resulting in increased morbidity and mortality [35,36]. At weaning, piglets are suddenly shifted to a solid diet and transferred to a new environment, which causes the gut microbiota to reassemble. Within a short period of time, the gut microbiota must develop and learn to adapt to the new nutrient source. A study by Wei et al. found that the relative abundance of *Prevotella* increased dramatically and rapidly after introducing solid plant-based feed: from 8.9% on the day of weaning to 17.6% on day 7 post-weaning [15]. The relative abundance of *Prevotella* is closely related to the consumption of plant polysaccharides and is enriched by plant polysaccharides [37]. Similarly, *Megasphaera* and *Blautia*, which are involved in carbohydrate digestion, also increased during the post-weaning period [15]. These bacterial changes will impact others and affect the entire gut ecosystem. For example, *Prevotella* supplants members of *Bacteroides*. Many studies indicated that subjects with a high relative abundance of *Prevotella* usually harbor a lower prevalence of *Bacteroides*, indicating that gut niche competition occurs between these two genera [38]. *Bacteroides* benefits their host by conducting immunomodulatory, metabolic, and trophic functions [39,40]. The beneficial effects of *Bacteroides fragilis* on the immune system are mediated by capsular polysaccharides, which positively impact the development of CD4^+^ T cells, affect Th1/Th2 balance, and induce regulatory T cells to secrete interleukin 10 (IL-10, a potent anti-inflammatory cytokine that protects the gut against pathogenic inflammation) [41]. Thus, the relative lower abundance of *Bacteroides* post-weaning may put the piglets at increased risk of intestinal disease [42].

In addition, Li et al. reported that the weaned pigs’ gut microbiota composition was characterized by the decreased proportion of *Alloprevotella* and *Oscillospira* and increased *Campylobacterales* [43]. *Alloprevotella* is an acetic and succinic acid producer, which plays a key role in maintaining the intestinal epithelial barrier and enhancing intestinal immune function [44]. Li et al. found that succinate upregulated the expression of tight-junction proteins (claudin-1, ZO-1, and ZO-2) and positively modulated inflammatory cytokine (IL-6 and IL-18) expression in the growing pig jejunum [45]. *Oscillospira*, a core microbiota member related to health, is a butyrate producer [46,47,48,49]. Butyrate not only acts as the dominant energy source for colonocytes but also prevents intestinal inflammation as well as systemic infection [50]. A decrease in *Oscillospira* levels has always been associated with inflammatory disease [48,51]. Taken together, decreased relative abundances of *Alloprevotella* and *Oscillospira* demonstrated that the gut lost certain anti-inflammatory properties after weaning. Furthermore, *Campylobacter* was one of the opportunistic pathogens causing acute bacterial diarrhea [52]. Hence, an increase in *Campylobacter* relative abundance may be one of the factors triggering post-weaning diarrhea.

### 2.2. The Importance of Maintaining a Healthy Gut

The animal GI tract is colonized by dynamic and complex microorganisms, known as the gut microbiota [53]. With the advent of next-generation sequencing, the characteristics of the swine gut microbiota have become clear. In general, the swine intestinal microbiota is predominated by two phyla, Firmicutes and Bacteroidetes, followed by Proteobacteria, Actinobacteria, and Spirochaetes, among others [15,16]. This general profile is relatively stable, although variations exhibit below the phylum level when the gut microbiota is disturbed.

Nowadays, the beneficial and functional roles of the gut microbiota in maintaining host health have been better identified by germ-free and fecal transplant studies [54,55]. Studies conducted by Tsai et al. and Cheng et al. found that fecal microbial transplantation was capable of improving the growth performance of both suckling and weaning pigs by modulating the gut microbiota [56,57]. In addition, antibiotics lack growth-promoting functions in germ-free animals [58]. In this section, we will briefly summarize the main functions of the gut microbiota.

#### 2.2.1. Nutrient Metabolism

The gut microbiota possesses the ability to produce various digestive enzymes, which facilitate energy extraction from complex carbohydrates [59]. The major end products of the fermentation of non-digestible carbohydrates by microbes are short-chain fatty acids (SCFAs) such as butyrate, propionate, and acetate [60]. SCFAs serve as energy sources for both host cells and intestinal bacteria and modulate host metabolic responses. They are involved in various host-signaling mechanisms and play a central role in inhibiting histone deacetylases and activating G-coupled-protein receptors [61].

Intestinal bacteria possess numerous enzyme genes that are associated with carbohydrate-degrading activities [59]. For example, *Bacteroides thetaiotaomicron* produces starch-degrading enzymes [62]. At the same time, enzymes from *Bacteroides ovatus* are characterized by hydrolyzing galactomannans, xylans, and mannans [63,64]. In addition, *Bifidobacterium spp.* are able to degrade high amylose starches [65].

The gut microbiota also has the ability to synthesize certain vitamins, such as vitamins K and B, which are important components in maintaining host health [66,67]. Examples of vitamin-producing bacteria are *Bifidobacterium*, *Bacteroides*, and *Enterococcus* [68].

#### 2.2.2. Immunomodulatory and Anti-Inflammatory Effects

The crosstalk between the gut microbiota and the immune system has been investigated tremendously. Microbial components and metabolites impact the immune system through neutrophil function modulation, neutrophil recruitment and activation, and T cell differentiation [69,70]. Extensive mouse-model studies have demonstrated that microbial reduction or absence greatly decreases the number of neutrophils and their progenitors, making the host more vulnerable to infection. As we discussed earlier, gut microbiota-released metabolites such as SCFAs could act as histone deacetylase (HDAC) inhibitors and therefore attenuate inflammatory responses by blocking nuclear factor-κB (NF-κB) signaling [71,72,73,74]. In addition, microbiota-derived aryl hydrocarbon receptor (AHR) ligands enhance regulatory T cells’ (Tregs) immunosuppressive activities during inflammation, which indirectly influences neutrophil recruitment and activation [75,76]. Moreover, bioactive amines (e.g., histamine), generated by commensal bacteria from amino acids, play a crucial role in regulating immune responses [77].

#### 2.2.3. Impacts of Gut Microbiota on Intestinal Barrier Function and Gut Structure

The intestinal barrier is the host’s first line of defense against potentially pathogenic bacteria and toxic molecules [78]. Mounting evidence indicates that commensal bacteria contribute to the structural and functional development of the gastrointestinal tract. For example, *Lactobacillus rhamnosus* GG-derived protein p40 inhibits cytokine-induced apoptosis of intestinal epithelial cells [79]. *Bacteroides thetaiotaomicron* is involved in desmosome maintenance at the epithelial villus by inducing the expression of SPRR2A (small proline-rich protein 2A) [80]. Another example of an intestinal bacteria that influences barrier function is *Akkermansia muciniphilia*, which regulates epithelial barrier permeability by increasing endocannabinoid levels [81].

The benefits of the microbiota in supporting gut structure and function can be easily appreciated from germ-free mice studies. Microbiota-lacking animals exhibit thin villi, weak peristalsis, decreased intestinal surface area, reduced villus capillary network, and prolonged cell cycle time [82].

## 3. The Effects of Nutritional Interventions on Swine Gut Health around Weaning

In order to overcome weaning stress, feed additives are commonly used in the swine industry to improve intestinal function, increase feed utilization, enhance immune response, prevent diseases, and improve growth rate [83]. Examples of additives used in swine diets include zinc, peptides, organic and inorganic acids, prebiotics, probiotics, plant extracts, essential oils, and yeast, as reviewed by Liu et al. [83]. Compared to other published reviews, this review includes more information about how feed additives impact the swine gut microbial ecology. This section will focus on several feed additives known to modulate the swine intestinal microbiota with some success; putative mechanisms and the growth performance results for each additive are also included. 

### 3.1. Zinc

#### 3.1.1. Pharmacological Role of Zinc

Nursery pigs usually require 80 to 100 ppm of zinc (Zn) to meet their requirement for growth [84]. Feeding pharmacological levels of inorganic Zn to nursery pigs is usually recommended to combat postweaning diarrhea and improve growth performance [85,86,87,88]. Hahn and Baker evaluated the effects of the additional of high levels (3000 ppm and 5000 ppm) of Zn from different sources on the growth performance of weanling pigs. They found that ZnO addition, regardless of level, significantly increased both daily gain and daily feed intake, whereas inconsistent growth performance response was observed when using ZnSO_4_. At the same time, they conducted a trial to determine whether 3000 ppm of dietary Zn from ZnO or ZnSO_4_ had positive effects on weaning pigs fed a high nutrient density diet (basal starting diet fortified with both plasma protein and fish meal and containing more dried whey). The results showed that only pigs fed 3000 ppm ZnO had significantly increased weight gain and voluntary feed intake. ZnSO_4_ addition did not elicit a performance response [89]. These results indicated that feed composition and different chemical forms of Zn might affect the response outcome. In addition, the duration of pigs on high Zn diets greatly impacts the growth performance. Carlson et al. determined that supplementing 3000 ppm of Zn as ZnO immediately after weaning for two weeks could accelerate the growth of both early (<14 d) and traditionally (>21 d) weaned pigs; however, the addition of Zn could not enhance growth if fed only during the first week after weaning [90]. It is possible that newly weaned pigs do not absorb enough Zn due to the low feed intake during the first week after weaning.

Compared with traditional ZnO, nano-size ZnO is smaller in size and has a larger surface area and higher bioavailability [91]. Pei et al. found that 450 ppm Zn from nano-ZnO could enhance the growth performance of weaned piglets as efficiently as high concentrations (3000 ppm) of conventional ZnO [92]. In the same study, they also suggested that 150 ppm nano-ZnO had positive influences on growth performance. However, a study from Li et al. reported that nano-ZnO at 120 ppm failed to improve the growth performance of weaned pigs [93]. The inconsistency of the results might be due to the different concentrations and methods of synthesis of nano-ZnO.

There are many possible mechanisms to explain why high levels of Zn have beneficial effects on the growth performance of weaned pigs. Studies have shown that Zn plays an important role in maintaining intestinal epithelial barrier function and even tightens “leaky gut” [94]. Hu et al. found that the benefits of Zn in the form of ZnO on intestinal integrity are related to its ability to decrease inflammatory gene expression by inhibiting the TLR4–MyD88 signaling pathways, which reduces the level of pro-inflammatory cytokines and chemokines [95]. The barrier protective role of Zn was also supported by a report that Zn deprivation could disassemble tight junction proteins and cause epithelial barrier dysfunction [96,97].

Weaning-induced villous atrophy in the small intestine could be reversed by Zn supplements [95]. Furthermore, high dietary levels of Zn have a positive impact on gut microbiota composition and stability. The weaning process dramatically affects gut microbiome composition [43]. Any disturbances in the stability of the gut microbiome could lead to the overgrowth of indigenous as well as exogenous pathogenic bacteria, which may lead to diarrhea. Katouli et al. suggested that 2500 ppm of Zn in pig diets could maintain the stability of the intestinal microbiota and high diversity of coliforms, which may compete with diarrheagenic strains for colonizing receptor sites [98]. More interestingly, high ZnO and antibiotics had similar effects on the gut microbiota of weaning piglets [99]. Both significantly increased the abundance of *Verrucomicrobia*, *TM7*, *Spirochaetes*, *Tenericutes*, and *Euryarchaeota* and reduced *Chlamydiae* in ileal digesta. Nevertheless, the impacts of high concentrations of ZnO on swine commensal microbiota remain controversial. Starke et al. reported that the supplementation of the piglet diet with high concentrations of ZnO was associated with an increase in *Megasphaera* and a decrease in *E. coli* and *Enterobacteriaceae*, whereas other researchers such as Højberg et al., Vahjen et al., and Wei et al. noticed opposite trends [16,100,101,102]. These inconsistent results might be related to differences between the studies, such as various weaning days, sampling timepoints, fecal collection from various intestinal segments and rectal swabs, and separate nursing facility environments. Although pharmacological levels of Zn are effective at controlling postweaning diarrhea, the environmental burden from excessive levels of Zn excretion from manure has led many countries to limit Zn usage as a feed supplement. For example, the European Union limits ZnO usage at 150 ppm in swine diet [103] and China restricts the use of ZnO to 1600 ppm [104].

Apart from ZnO, other organic forms of Zn such as Zn-methionine and Zn-lysine are available for inclusion in diets at lower levels due to their greater bioavailability compared to ZnO [105]. For example, Ward et al. reported that the inclusion of 250 ppm Zn-methionine in nursery starter diets had beneficial effects equivalent to 2000 ppm of Zn from ZnO when 160 ppm of Zn from ZnSO_4_ was added in both diets [106]. However, this finding is in contrast to those of Hollis et al., who reported that supplementing 500 ppm Zn from several organic sources (Zinc polysaccharide complex, Zinc proteinate, Zinc amino acid complex, Zinc amino acid chelate, and Zinc methionine) was not as effective at stimulating growth rate and feed efficiency as high levels of ZnO (2000 ppm) [107]. Taken together, these findings suggest that further research is needed to investigate and determine the most effective condition and dose for the inclusion of organic Zn in piglet diets.

#### 3.1.2. Antimicrobial Activity of Zinc

**Figure 2:**
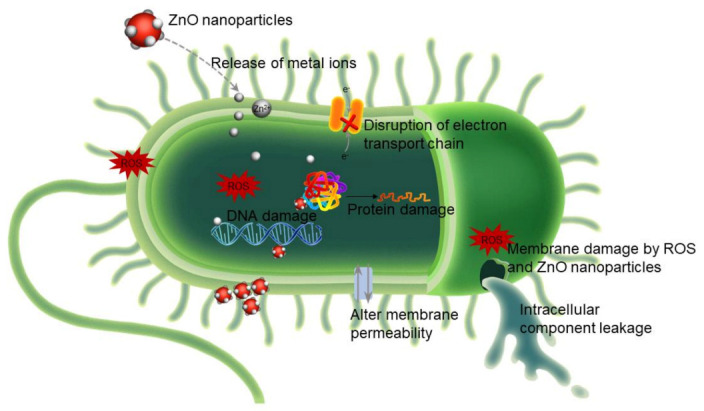
Schematic representation of probable mechanisms involved in ZnO antimicrobial activity. (i) The generation of reactive oxygen species (ROS), (ii) release of Zn^2+^ ions, and (iii) alteration of bacterial membrane permeability.

A bacterial infection is one of the most serious animal health issues. ZnO is widely known for its antibacterial properties, which have been investigated largely with both Gram-positive and Gram-negative bacteria. The main conclusions of these investigations can be summarized as follows: (1) ZnO shows a size-dependent toxic effect on bacteria and smaller ZnO particle size exhibits better antimicrobial activity [108,109,110]; (2) the antibacterial activity is related to the particle surface area and concentration, and the higher concentration and larger surface area have better antibacterial activity [111]; (3) the antibacterial activity of ZnO decreased with increasing heating temperature [112]; and (4) several proposed mechanisms that explained ZnO antibacterial activity are illustrated in Figure 2 and described below.

(i) Reactive oxygen species (ROS) generation.

Studies by Sawai et al. and Lipovsky et al. highlighted that ROS was the main mechanism for ZnO antibacterial activity [113,114]. The reactive species are hydrogen peroxide (H_2_O_2_), hydroxide (OH^−^), and superoxide anion (O^2^). The toxicity of these species involves the destruction of cellular components, such as lipids, DNA, and proteins.

(ii) Zinc ion (Zn^2+^) release.

The release of zinc ions in a medium containing ZnO and bacteria plays an important role in the toxic effect of ZnO particles [115]. Metal ions enter bacterial cells, followed by a reduction in ATP levels and disruption to DNA replication.

(iii) Changes in bacterial membrane permeability.

Some researchers proposed that there are electrostatic forces that serve as a powerful bond between metal oxide nanoparticles and bacteria. As a result, the cell membrane is damaged. Stoimenov et al. demonstrated that the opposite charge brings the bacteria and metal oxide nanoparticles together [116]. Zhang et al. also referred to an electrostatic phenomenon arising when *E. coli* was treated with ZnO due to the negative charge of the bacterial cell wall and positive charge of ZnO in a water suspension [117]. Such direct interaction may be attributed to bacterial membrane disorganization. As the ZnO nanoparticles precipitated on the bacteria exterior, the cell internalized these nanoparticles, which caused the cellular contents to leak out [118].

### 3.2. Peptides

#### 3.2.1. Functional Properties of Bioactive Peptides

Bioactive peptides, which possess multiple biological functions, have many potential beneficial effects on health [119,120]. A wide variety of natural sources, including animals, plants, and microorganisms, could produce antimicrobial peptides (AMP). These peptides show antimicrobial activities against pathogens, including bacteria, fungi, and viruses [121,122]. The direct killing mechanisms of AMP can be divided into membrane targeting and non-membrane targeting. The membrane targeting AMPs can be further divided into receptor-mediated and non-receptor-mediated interactions [123]. Three models have been proposed to describe how AMPs penetrate microbial membranes: the barrel-stave model [124], the carpet model [125], and the toroidal-pore model [126]. For non-membrane targeting mechanisms, AMPs can either target the bacterial cell wall to inhibit cell wall synthesis or have intracellular targets to inhibit protein and nucleic acid synthesis and to disrupt enzymatic and protein activity [127,128,129,130]. In addition to killing bacteria directly, bioactive peptides exert immunomodulatory functions to enhance microbial killing and reduce and control inflammation. In an infection, peptides activate and recruit immune cells to the site of infection [131,132,133]. Moreover, bioactive peptides could decrease pro-inflammatory chemokine expression and consequently suppress inflammation [132,134]. 

#### 3.2.2. Peptide Absorption and Utilization in Animal Nutrition

Protein is an essential macronutrient for animal growth and reproduction. It needs to be degraded into amino acids or small peptides to be absorbed by the intestine and transported in the blood [135,136]. Evidence suggests that amino acids in peptide form are absorbed more readily and rapidly than free amino acids [137,138]. Thus, peptides derived from animals, plants, and microorganisms with well-defined physiological roles are incorporated into commercially available products. Scientific data suggest that peptides have promising applications in animal nutrition. Pigs fed a diet containing spray-dried porcine intestine hydrolysate for two weeks after weaning had better growth performance than those fed control diets containing spray-dried plasma or dried whey [139,140]. A positive carry-over effect was detected [140], which was likely associated with increased intestinal villus area as well as an improved digestibility and absorption rate of dietary nutrients [141]. Moreover, Opheim et al. reported that adding hydrolyzed Atlantic salmon viscera in a broiler diet, at inclusion levels of 5% and 10%, resulted in better growth performance compared with either a plant protein-based diet or a fishmeal control diet [142].

Tang et al. found that feeding AMPs (Lactoferrampin and Lactoferricin) derived from bovine milk improved growth performance and decreased diarrhea rate of weaning pigs infected by *E. coli* [143,144]. Xiong et al. observed that dietary AMP mixture (2.0 or 3.0 g/kg) supplementation (lactoferrin, cecropin, defensin, and plectasin) improved the average daily gain of weaned pigs reared on five different commercial farms and pigs fed with 2.0 g/kg AMP had greater daily gain than those fed with 3.0 g/kg AMP [145]. However, Yoon et al. reported that the average daily gain increased with increasing levels of AMP-A3 (60 and 90 mg/kg) or AMP-P5 (40 and 60 mg/kg) [146,147]. These findings were in contrast to a study by Shan et al., who did not observe improvement in daily gain when pigs were fed with 1.0 g/kg of lactoferrin [148]. The differences between the results may be related to the types and doses of AMPs applied in the experiments. 

The beneficial effects of AMPs on intestinal development have also been reported in weaned pigs. A study from Xiao et al. found that AMP supplements were able to alleviate intestinal injury induced by mycotoxin deoxynivalenol in weaning pigs [149,150]. Wang et al. observed that lactoferrin increased villus height and decreased crypt depth of the intestine, directly affecting nutrient absorption and contributing to improved growth performance [151]. Studies by Wang et al. and Tang et al. revealed that adding the peptides lactoferrin or lactoferramoin-lactoferricin in nursery diets increased some beneficial bacteria such as *Lactobacillus* and *Bifidobacterium*, and decreased the pathogenic bacteria *E. coli* and *Salmonella* in the small intestine [151,152]. Wu et al. also reported that cecropin AD enriched *Lactobacillus* and reduced diarrhea incidence in weaned piglets challenged with *E.* coli [153]. In addition, Poudel et al. found that a peptide-based additive could accelerate the maturation of swine gut microbiota [154].

### 3.3. Organic Acids

The exact mode of action of dietary organic acids remains unknown, but several mechanisms have been proposed. Their benefits on animal growth performance are believed to be associated with reducing gastrointestinal pH, inhibiting pathogenic bacteria, serving as an energy source, and facilitating mineral utilization [155].

#### 3.3.1. Lowering Gastrointestinal pH

Protein digestion begins in the stomach with the help of pepsin, which is a potent enzyme utilized for breaking down proteins into smaller peptides [156]. Pepsin activity depends on acidic environments. Piper and Fenton reported that pepsin exhibits maximum activity in the pH range of 1.5–2.5. A pH outside this range could reduce or even inhibit enzyme activity [157].

Newly weaned pigs generate insufficient levels of gastric acid, which results in high stomach pH and consequently reduces protein digestion [158]. Many experiments have been conducted to examine the effects of various acidifiers on the reduction of the stomach and gastrointestinal tract pH of nursery pigs. However, the results were inconsistent. For example, Radcliffe et al. found stomach digesta pH was significantly decreased when pigs were fed citric acid [159]. Scipioni et al. reported a nonsignificant decrease in the stomach and jejunal pH by feeding citric acid in a complex diet [160]. Similar results were found in later work conducted by Burnell et al. [161]. Further research is needed to clarify these inconsistent results.

#### 3.3.2. Inhibition of Pathogenic Bacteria

Dietary organic acids lower stomach and intestinal pH, thus providing a less hospitable environment for pathogenic microbes. The pH in the stomach has two main functions: (1) stimulates digestive enzyme secretion, and (2) provides a barrier against the entry of foreign bacteria into the small intestine. As previously mentioned, early weaned piglets have a limited capacity to digest protein. These undigested proteins could promote microbial fermentation, leading to the proliferation of harmful bacteria in the gastrointestinal tract [162]. Thus, lowering the pH in the gastrointestinal tract is important for weanling pig health. A study by Thomlinson and Lawrence revealed that dietary lactic acid decreased stomach pH and delayed the multiplication of enterotoxigenic *E. coli* in weaned pigs [163]. In addition, adding butyric acid to chicken diets protected broilers from *Salmonella* infection [164,165]. Furthermore, these acids had no antimicrobial effects on pH-insensitive beneficial bacteria such as *Lactobacilli* and *Bifidobacterium spp.* [155]. A recent study by Wei et al. conducted in weaning pigs indicated that pigs fed an organic acid mixture had more diverse microbiota and produced more potential beneficial bacteria such as *Turicibacter*, *Blautia*, and *Oscillospira* and decreased proportions of *Sarcina* and *Veillonella*, which are linked to various inflammatory diseases [15].

#### 3.3.3. Energy Source

Organic acids are commonly used in pig nutrition, where they serve as a direct energy source, and some are important players in metabolic pathways and cycles. It has been suggested that pigs can utilize fumaric acid as an energy source as efficiently as glucose [166]. Blank et al. pointed out that there is a possibility that fumaric acid, as a readily available energy source, may have a local trophic effect on the small intestine mucosa and lead to an increase in the absorptive surface area and capacity of the small intestine due to the faster recovery of the gastrointestinal epithelial cells after weaning [167].

#### 3.3.4. Beneficial Effects of Organic Acids on Swine

The benefits of organic acids in enhancing nutrient digestibility and modulating intestinal microbiota should improve growth performance in weanling pigs. Falkowski and Aherne demonstrated that dietary fumaric acid or citric acid improved the average daily gain and feed conversion ratio of weanling pigs [168]. Likewise, Giesting and Easter found that there was a linear increase in feed efficiency and daily gain when weanling pigs were supplied with increasing levels of fumaric acids [169]. Various other acidifiers such as benzoic acid and butyric acid also showed positive effects on growth performance [170,171,172,173]. In contrast, Ahmed observed that the acidifier blend (formic acid, propionic acid, lactic acid, phosphoric acid, and SiO2) negatively impacted growth performance parameters of weaned pigs [174]. In addition, the effects of organic acid on nutrient digestibility remained controversial. For instance, fumaric acid had no or even adverse effects on the ileal digestibility of amino acids [175,176]. However, a positive effect on the apparent total tract digestibility of protein was reported [177]. Besides, it has been reported that dietary benzoic acid improved the apparent ileal digestibility of total nitrogen in weanling pigs [170,178]. Factors such as diet buffering capacity as well as acidifier types and concentrations seem to contribute to these inconsistent results. Further investigations are thus needed to clarify the modes of action of acidifiers and to outline the specific conditions under which they are most effective for various available acidifiers applied in weaning piglets.

### 3.4. Probiotics

#### 3.4.1. Probiotics in Swine Nutrition

Probiotics are defined as live microorganisms, which, when administered in adequate amounts, confer a health benefit on the host [179]. Azad et al. thoroughly reviewed probiotics and their biological effects [180]. Members of the genera *Lactobacillus*, *Bifidobacterium*, and *Bacillus* with probiotic properties are the most common microorganisms applied in the swine industry, but not exclusively, as a growing number of probiotic microorganisms have been discovered and are available for swine production. The beneficial effects of probiotics such as *Lactobacillus reuteri* and *Bacillus licheniformis* on the growth performance of weanling pigs have been reported [181,182,183]. Giang et al. observed that the inclusion of 0.06% probiotic complex (*Enterococcus faecium* 6H2, *Lactobacillus acidophilus* C3, *Pediococcus pentosaceus* D7, *L. plantarum* 1K8, and *L. plantarum* 3K2) in diets had positive effects on average daily gain, average daily feed intake, and feed/gain ratio during the first two weeks post weaning, whereas no effects were found in the following period [184]. Likewise, Huang et al. demonstrated that dietary 0.1% *Lactobacillus* complex (*L. gasseri*, *L. reuteri*, *L. acidophilus*, and *L. fermentum*) supplementation improved the average feed intake of piglets during the first two weeks after weaning [185]. It is possible that gastrointestinal bacteria in newly weaned piglets were vulnerable and could be easily changed by external factors such as probiotics. Therefore, the positive effects of probiotics on growth performance were only observed in the first two weeks after weaning. However, different *Lactobacillus* complex and inclusion levels may cause different results. For example, Zhao et al. reported that the inclusion of *L. reuteri* and *L. plantarum* complex (0.1% or 0.2%) in diets had no effects on average daily gain, average daily feed intake, or feed/gain in weaning pigs [186]. In fact, a reduction in feed efficiency was reported by Frantz et al. as a result of a 0.1% *Lactobacillus* diet [187].

#### 3.4.2. Probiotics as Modulators of Swine Gut Microbiota

In several recent studies, the supplementation of piglet diet with probiotics has been associated with an increase in *Lactobacillus* or *Bifidobacterium spp*. and a decrease in *E. coli* [188,189,190,191]. These changes possibly benefit intestinal health, which can lead to improved growth performance. Zhang et al. found that oral administration of the *Bacillus licheniformis*-*B. subtilis* mixture modulated the gut microbiota of weaned pigs by enriching certain members of *Clostridium*, *Lactobacillus*, and *Turicibacter* [192]. In addition, dietary *Bacillus subtilis* could enrich bacteria with high fiber fermentation efficacy [193]. Besides, Shin et al. indicated that gut microbial diversity and richness (Simpson, Shannon, ACE, and Chao 1) were higher in pigs fed the probiotic microbe *L. plantarum* JDFM LP1 than those fed the basal diet [194]. However, Wang et al. found that dietary *L. plantarum* PFM105 had no influences on Shannon, Chao 1, observed species, and ACE, although the Simpson index was increased. In addition, overconsumption of probiotics may disturb the gut microbial ecosystem and increase the risk of enteric disease [192,195,196]. Overall, these results suggest that the benefits of probiotics on the gut are strain- and dosage-dependent.

## 4. Conclusions

To mitigate the negative effects of weaning, nutrition intervention strategies are selected to increase nutrient digestibility, enhance intestinal health, and prevent or mitigate disease. In response to intervention, many notable shifts in the swine gut structure and microbial communities were detected. These changes benefit gut health and probably contribute to improved growth performance.
